# Targeting the EGF receptor family in non-small cell lung cancer—increased complexity and future perspectives

**DOI:** 10.20892/j.issn.2095-3941.2022.0540

**Published:** 2022-12-05

**Authors:** Tobias Boch, Jens Köhler, Melanie Janning, Sonja Loges

**Affiliations:** 1 DKFZ-Hector Cancer Institute at the University Medical Center Mannheim, Mannheim 68135, Germany; 2 Division of Personalized Medical Oncology (A420), German Cancer Research Center (DKFZ), Heidelberg 69120, Germany; 3 Department of Personalized Oncology, University Medical Center Mannheim, Medical Faculty Mannheim, Heidelberg University, Mannheim 68135, Germany

**Keywords:** Oncology, NSCLC, EGFR, HER2, HER3, HER4

## Abstract

Lung cancer remains the leading cause of cancer-associated mortality worldwide, but with the emergence of oncogene targeted therapies, treatment options have tremendously improved. Owing to their biological relevance, members of the ERBB receptor family, including the EGF receptor (EGFR), HER2, HER3 and HER4, are among the best studied oncogenic drivers. Activating EGFR mutations are frequently observed in non-small cell lung cancer (NSCLC), and small molecule tyrosine kinase inhibitors (TKIs) are the established first line treatment option for patients whose tumors bear “typical/classical” EGFR mutations (exon 19 deletions, L858R point mutations). Additionally, new TKIs are rapidly evolving with better efficacy to overcome primary and secondary treatment resistance (e.g., that due to T790M or C797S resistance mutations). Some atypical EGFR mutations, such as the most frequent exon 20 insertions, exhibit relative resistance to earlier generation TKIs through steric hindrance. In this subgroup, newer TKIs, such as mobocertinib and the bi-specific antibody amivantamab have recently been approved, whereas less frequent atypical EGFR mutations remain understudied. In contrast to EGFR, HER2 has long remained a challenging target, but better structural understanding has led to the development of newer generations of TKIs. The recent FDA approval of the antibody-drug conjugate trastuzumab-deruxtecan for pretreated patients with HER2 mutant NSCLC has been an important therapeutic breakthrough. HER3 and HER4 also exert oncogenic potential, and targeted treatment approaches are being developed, particularly for HER3. Overall, strategies to inhibit the oncogenic function of ERBB receptors in NSCLC are currently evolving at an unprecedented pace; therefore, this review summarizes current treatment standards and discusses the outlook for future developments.

## Introduction

### The ERBB family of receptors (EGFR, HER2, HER3 and HER4) in non-small cell lung cancer (NSCLC)

Lung cancer remains the leading cause of cancer-associated mortality worldwide^1^. NSCLC accounts for 85% of all lung cancer cases and is a genetically heterogeneous disease. During the past 2 decades, however, oncologists have learned how to harness important driver oncogenes as therapeutic targets; their inhibitors, primarily tyrosine kinase inhibitors (TKIs), have revolutionized treatment paradigms and significantly improved patient outcomes beyond those achievable with standard chemotherapy.

The ERBB family of receptors, also known as the epidermal growth factor receptor (EGFR) family, has become an important therapeutic target in NSCLC. This family includes the epidermal growth factor (EGF) receptor (EGFR, ERBB1/HER1), HER2 (ERBB2), HER3 (ERBB3) and HER4 (ERBB4). These transmembrane receptors are normally activated by homo- or heterodimerization after ligand-binding, but gain-of-function mutations in the tyrosine kinase domain, e.g., of EGFR, or gene amplifications render them nearly ligand independent^2^. Although several generations of EGFR TKIs have been clinically approved, targeting HER2 and HER3 has long been challenging because HER2 lacks a natural ligand, thus making receptor heterodimerization crucial for its activation, and because HER3 has impaired kinase activity^3^. HER4 is expressed at low levels in NSCLC, and the importance of HER4 mutations remains incompletely understood to date.

Overall, studies have suggested that more than 50% of advanced stage lung adenocarcinomas bear genetic alterations with therapeutic relevance. With the emergence of new drug classes including antibody-drug conjugates (e.g., trastuzumab-deruxtecan) and monoclonal (bispecific) antibodies (e.g., amivantamab) in addition to “classical” TKIs, the field of EGFR family targeting is rapidly evolving with an increasingly complex drug landscape, as reviewed herein.

## The epidermal growth factor receptor (EGFR and ERBB1)

**Figure 1:**
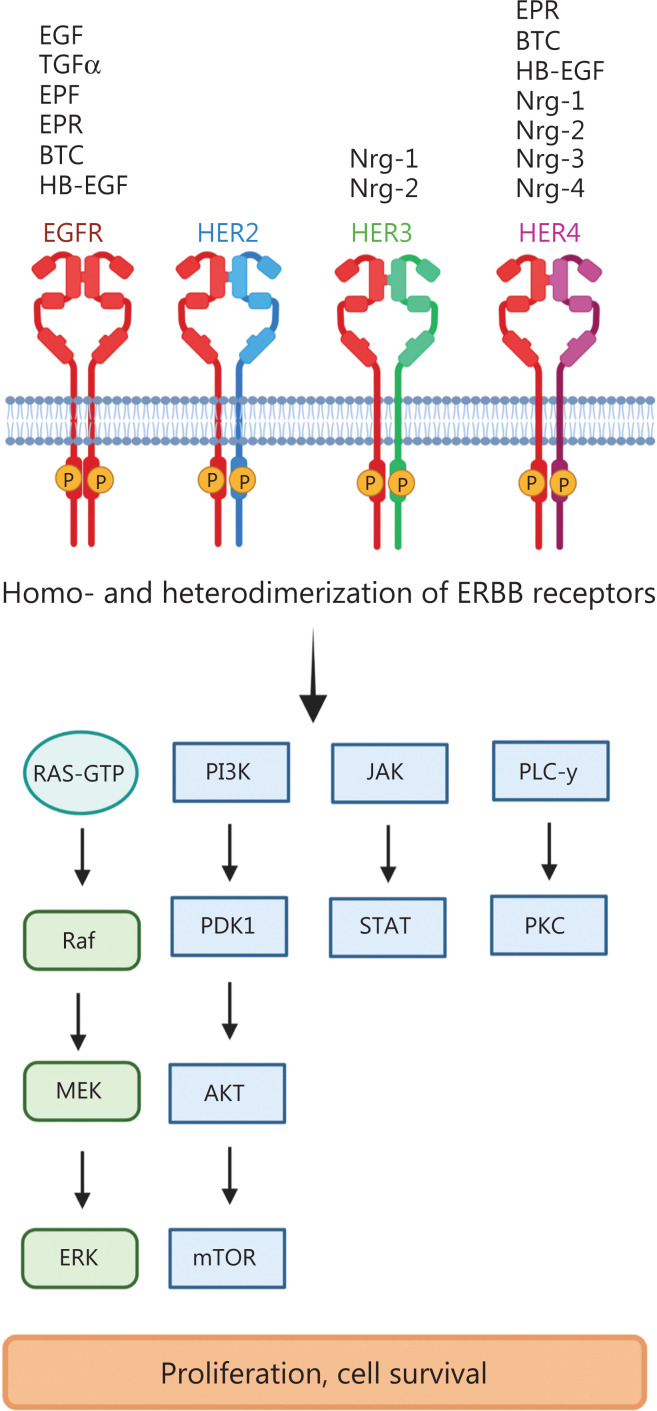
ERBB receptor-dependent signaling. The binding of growth factors to ERBB/HER family members induces the phosphorylation of tyrosine kinases and subsequent activation of downstream signaling pathways such as Ras-RAF-MEK-ERK (extracellular signal-regulated kinases); PI3K (phosphoinositide 3-kinase)–PDK1 (3-phosphoinositide-dependent protein kinase 1), AKT (protein kinase B)–mTOR (mammalian target of rapamycin); PLC γ (phospholipase C γ), PKC (protein kinase C) and JAK (Janus kinase)-STAT (signal transducers and activators of transcription). EGF (epidermal growth factor), TNFα (tumor necrosis factor α), EPF (extracellular protein factor), EPR (epiregulin), BTC (betacellulin), HB-EGF (heparin-binding epidermal growth factor), Nrg1–4 (neuregulin 1–4). Created with BioRender.com.

The EGFR was the first tyrosine kinase receptor to be discovered and subsequently associated with cancer^4^. The *EGFR* gene consists of 28 exons, which encode 2 EGF-binding domains (exons 5–7 and 13–16), a transmembrane domain (exon 17), a tyrosine kinase domain (exons 18–24) and an autophosphorylation domain (exons 25–28). EGFR’s 2 cognate ligands, EGF and transforming growth factor alpha (TGFα), after receptor binding, activate multiple downstream signaling pathways, including the mitogen-activated protein kinase (MAPK), phosphoinositide 3-kinase (PI3K)/AKT, protein kinase C (PKC) and Janus kinase/signal transducers and activators of transcription (JAK/STAT) signaling pathways, which increase cancer cell proliferation, pro-survival functions and apoptotic resistance^5^ (**Figure 1**).

### Classical/typical EGFR mutations in NSCLC

**Figure 2:**
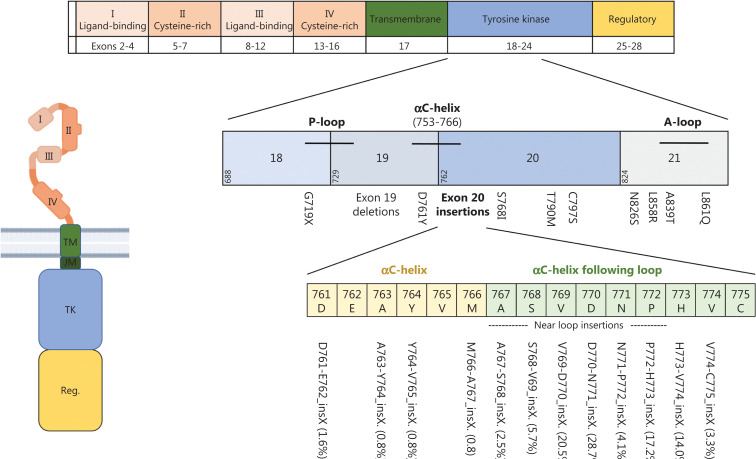
EGFR protein domains and most frequent mutations. The extracellular domain of EGFR is composed of 2 extracellular ligand-binding domains (I and III) as well as 2 cysteine-rich domains (II and IV) that support dimer formation. It is linked *via* a single-α-helix transmembrane domain (TM) to the intracellular domain, which consists of a tyrosine kinase domain (TK) and a carboxy-terminal regulatory domain (Reg.). Activating gene mutations affecting the tyrosine kinase domain occur in exons 18–21. Exon 19 deletions and L858R point mutations are so-called “classical mutations” and occur in 70%–90% of cases. Atypical mutations occur primarily in exon 20. Beyond the common T790M mutation, exon20 insertion mutations are of particular relevance. Frequencies of different exon20 insertion mutations are displayed in brackets. Created with BioRender.com.

The overall prevalence of EGFR mutations in NSCLC varies by ancestry, ranging from 10% to 20% in white populations and as high as 50% in Asian populations; higher frequencies are also observed in women and people who have never smoked^6^. Gain-of function mutations in exon 21 (mostly L858R) or deletions in exon 19 are the largest group of all EGFR mutations (70%–90%) and are designated “classical” or “typical” EGFR mutations^7,8^. They are found near the ATP-binding pocket and decrease the receptor’s affinity for ATP, thus subsequently leading to ligand-independent receptor activation and increased migration, proliferation and apoptotic resistance of cancer cells^2,8^. **Figure 2** shows the EGFR protein domains and genomic localization of different EGFR mutations.

Patients with NSCLC bearing typical EGFR mutations have better outcomes when they are treated with approved first generation (reversible inhibitors erlotinib and gefitinib), second generation (irreversible inhibitors afatinib and dacomitinib) or third generation (T790M targeting inhibitors osimertinib and lazertinib) EGFR TKIs than with chemotherapy (summarized in **Table 1**). Therefore, frontline EGFR TKI treatment has become the standard of care for patients with NSCLC with classical EGFR mutations. Second generation TKIs have been shown to be superior to first generation TKIs in terms of PFS^9,10^. Third generation TKIs have been developed to target the T790M mutation alongside the L858R point mutation or exon 19 deletion. The FLAURA trial has indicated superiority, including an OS benefit, of osimertinib over gefitinib or erlotinib as a first line treatment in patients with classical EGFR mutations^11,12^. Despite some differences in global TKI application because of differences in drug accessibility and reimbursement policies, osimertinib is usually the preferred TKI for these patients, owing to its efficacy and ability to penetrate the CNS. Recently, osimertinib was also approved in the adjuvant therapy setting for patients with stage IB-IIIA NSCLC (UICC 7^th^ edition) whose tumors bear classical EGFR mutations^13^. Thus, beyond metastatic disease, EGFR-targeted therapies have also been used in the treatment of early stage NSCLC. **Table 1** summarizes the clinical outcomes of patients with NSCLC bearing L858R and exon 19 deletions receiving different EGFR TKIs as first line treatments.

**Table 1:** Clinical outcomes of patients with NSCLC bearing L858R and exon 19 deletions after first line treatment with the indicated EGFR TKIs

Study	Phase	Patients (*n*)	Drugs	mPFS (months)	mOS (months)	Ref.
• Park et al., 2016 (Lux-Lung 7)	Phase IIB	159	Gefitinib	10.9	25.5	^10^
		160	Afatinib	11.0	27.9	^10^
• Wu et al., 2017 (Archer1050)	Phase III	225	Gefitinib	9.2	26.8	^183^
		227	Dacomitinib	14.7	34.1	^183^
• Soria et al., 2018 (FLAURA)	Phase III	277	Gefitinib/erlotinib	10.2	31.8	^12^
		278	Osimertinib	18.9	38.6	^12^

### Development of resistance to EGFR TKIs in patients with classical/typical EGFR mutations

**Figure 3:**
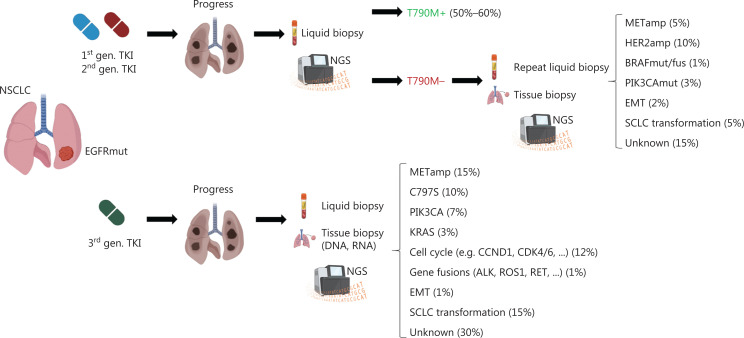
Diagnostic workflow after disease progression and most common resistance mechanisms to EGFR tyrosine kinase inhibitors. In patients who experience progression after treatment with first or second generation EGFR TKIs, a liquid biopsy with next-generation sequencing should be performed to determine the most common resistance mutation, T790M. If T790M negativity is found, liquid biopsy should be repeated, and additional tissue biopsies should be obtained if clinically feasible. In patients progressing after initial treatment with third generation TKIs (such as osimertinib), early tissue biopsy and additional liquid biopsy should be performed. The most common resistance mechanisms, such as MET amplification (METamp) or small-cell lung cancer (SCLC) transformation, are listed. MET (mesenchymal-epithelial transition), HER2 (human epidermal growth factor receptor2), BRAF (v-raf murine sarcoma viral oncogene homolog B1), PIK3CA (phosphatidylinositol-4,5-bisphosphate 3-kinase catalytic subunit α), EMT (epithelial-mesenchymal transition), KRAS (Kirsten rat sarcoma virus), CCND1 (cyclin D1), CDK 4/6 (cyclin-dependent kinase 4/6), ALK (anaplastic lymphoma kinase), RET (rearranged during transfection). Created with BioRender.com.

Despite the therapeutic success of EGFR TKIs in patients with NSCLC with classical EGFR mutations, the imposed selection pressure inevitably leads to treatment resistance. Resistance mechanisms fall into different categories and consist of on-target EGFR kinase domain mutations such as T790M, C797S or *EGFR* gene amplifications; activation of bypass signaling *via MET* amplification; trans-differentiation into small cell histology; or mutations affecting other driver oncogenes (e.g., BRAF). Interestingly, resistance mechanisms differ depending on the specific EGFR TKI. Although T790M mutations can arise *de novo* and subsequently hinder drug binding, they also occur in 50%–60% of patients treated with first- or second-generation EGFR TKIs. HER2 (10%) and MET (5%) amplifications, resistance mutations in BRAF (1%) or PIK3CA (3%), epithelial-mesenchymal transition (2%) or trans-differentiation into small-cell lung cancer (5%) are less frequently observed. In 15% of cases, the underlying resistance mechanism is unknown^14^. A schematic diagnostic workflow and summary of resistance mechanisms after TKI treatment are shown in **Figure 3**.

In contrast, after osimertinib treatment, on-target resistance mutations (e.g., C797S) occur in only 10% of patients, whereas MET amplifications are the more frequent resistance mechanism (~15%). Alterations in PIK3CA (7%), BRAF (3%), KRAS (3%) and cell cycle genes (12%), as well as gene fusions (e.g., involving RET) (1%) are important bypass-signaling pathways (**Figure 3**). Notably, at least 30% of resistance mechanisms remain currently unknown, thus indicating the need for deeper understanding of the biology of osimertinib resistance^15^.

### Strategies to overcome osimertinib resistance

Although T790M mutations arising *de novo* or induced by treatment (e.g., first- or second-generation EGFR TKIs), to date, can be targeted only with osimertinib or lazertinib (approved in South Korea only)^9^, promising strategies have emerged in recent years to treat specific resistance mechanisms that occur during osimertinib treatment. For on-target resistance mutations (e.g., C797S), several fourth generation EGFR TKIs (e.g., BLU-945, EAIO45, JIN-A02 and BBT176) are currently in development. These TKIs inhibit classical EGFR mutations, as well as T790M plus C797S. However, only preclinical and/or phase I data are available for these drugs to date^16,17^. For resistance-mediating MET amplification, an interesting treatment option is amivantamab, a bi-specific anti-EGFR/anti-MET antibody. Amivantamab has already received regulatory approval for patients with NSCLC with exon 20 insertions^18^, and it is currently being investigated together with lazertinib (a third generation EGFR inhibitor approved in Korea for EGFR T790M^+^ patients as a second line treatment^19^) in the multi-cohort basket trial CHRYSALIS-2 (NCT04077463). Of note, the amivantamab/lazertinib combination has been found to induce an ORR of 36% in patients pretreated with osimertinib and platinum-based chemotherapy^20^. The toxicity profile of this combination is acceptable and has shown no new safety concerns except the already known adverse events including infusion reactions (amivantamab) and dermatitis/stomatitis (EGFR TKI)^20^. The ongoing MARIPOSA trial (NCT04487080) is testing this combination in the frontline setting *vs.* osimertinib and lazertinib monotherapy. Given its promising efficacy and tolerability as a third line treatment, this treatment regimen may be particularly attractive in this setting if regulatory approval is granted. Rarely, fusions involving the *RET* gene have been reported as a resistance mechanism to Osimertinib, and a combination of erlotinib or osimertinib with the RET inhibitor selpercatinib [pralsetinib is another U.S. Food and Drug Administration (FDA) and EMA approved RET inhibitor option] has been found to be effective^21,22^. Overall, the best strategy appears to involve adding a resistance mechanism targeting drug to the EGFR inhibitor if the drug combination is well tolerated. If mutations affecting proteins downstream of EGFR, such as the BRAFV600E mutation, are present, on-label treatment with approved drugs, in this case dabrafenib and trametinib, is recommended^23^.

### Immunotherapy in patients with classical/typical EGFR mutations

Three independent phase III registration trials have indicated that mono-immunotherapy is inactive in the second line setting for patients with classical EGFR mutations^24–26^. Because similar results have been observed for anaplastic lymphoma kinase (ALK)-driven NSCLCs^27^, which are also more frequent in people who have never smoked or who are light smokers, this phenomenon is probably due to the low tumor mutational burden and low amount of neoantigens accompanying an immunologically cold tumor microenvironment^28^. The lack of immunotherapy efficacy as a second line treatment has led to the exclusion of patients with EGFR mutant NSCLC from most frontline registration trials with PD(L)1-blocking antibodies. However, in the IMPOWER 150 trial, the quadruplet combination of chemotherapy, bevacizumab and atezolizumab has been found to be effective in patients with typical EGFR mutations, and has been approval for post-TKI treatment, owing to the overall low chemotherapy efficacy after TKI failure^29^.

### Atypical/uncommon EGFR mutations

Despite rapid progress in targeting classical EGFR mutations, the biology of uncommon/atypical EGFR mutations is less well understood. Atypical EGFR mutations represent 10%–30% of all EGFR mutations, and occur alone or in combination with other (typical or atypical) EGFR mutations. In most cases, atypical EGFR mutations arise in exon 20 either as point mutations, mostly T790M (19%), or insertions (21%–26%)^7,30^ (**Figure 2**). Exon 20 mutations are highly heterogeneous and share structural similarities with HER2 exon 20 alterations and therefore are discussed separately herein.

Overall, only limited prospective data are available for patients with atypical EGFR mutations. In a pooled retrospective analysis of the LUX-Lung 2 (single arm afatinib), 3 and 6 trials (randomizing patients to afatinib *vs.* chemotherapy), patients with atypical EGFR mutations were categorized into 3 groups: group 1, with point mutations or duplications in exons 18–21; group 2, with *de novo* T790M mutations; and group 3, with exon 20 insertions^31^. Overall, groups 2 and 3 did not derive meaningful benefit from afatinib, in agreement with the lack of efficacy of second-generation EGFR inhibitors in patients with these alterations. In group 1, the EGFR L861Q mutation was the most frequent subtype (44%), followed by G719X (39%) and S768I (21%), and the median PFS for G719X and S768I mutations was longer than that for the L861Q mutation (13.8 and 14.7 months *vs*. 8.2 months, respectively)^31^. To provide deeper insight into the efficacy of different treatment modalities (EGFR TKIs, and chemo- and immunotherapy), Janning et al.^30^ have recently analyzed the largest dataset of patients (*n* = 234) with atypical EGFR mutations to date, on the basis of National Network Genomic Medicine (nNGM) data. In this dataset, exon 20 insertions (exon20ins) were the most frequent alterations (26.5%), but very rare point mutations represented a comparably large group (26.1%). Patients were categorized into 3 groups on the basis of mutations: (1) G719X, S768I, E709X, L861Q and combinations of those mutations with classical EGFR mutations; (2) exon 20 insertions; and (3) very rare point mutations as well as exon 18 deletions, exon 19 insertions and complex mutations. This study confirmed that patients in group 1 benefitted more from EGFR TKIs than from chemotherapy (PFS 6.6 months *vs*. 5.0 months); that, although the subgroups were small, afatinib was superior to erlotinib, gefitinib and osimertinib; and that immunotherapy was less efficacious overall. A major finding of this analysis was that group 3 patients with very rare EGFR mutations benefitted from TKI treatment, and the largest PFS benefit was observed for gefitinib. This study has also proposed a novel classification system for atypical EGFR mutations and reported the clinical activity of different TKIs^30^.

To address the heterogeneity of EGFR mutations, Robichaux et al.^7^ have developed predictive *in silico* models for TKI sensitivity and identified 2 subgroups of atypical EGFR mutations, on the basis of structural information: (1) classical-like mutations, which occur far from the ATP-binding pocket and therefore are predicted to have no or little effect on EGFR TKI drug binding and (2) mutations within the ATP-binding pocket or C-terminus of the of the C-helix (so-called PACC mutations, including S768I and G719X), which have been predicted to be P-loop and C helix-compressing, and to interfere with simertinib but not second-generation TKI binding, as subsequently confirmed with *in vitro* models and in larger clinical datasets^32^. This retrospective analysis therefore also suggests that patients with NSCLC with PACC mutations derive a higher benefit from afatinib than other EGFR TKIs. Osimertinib achieved a PFS of 8.2 months in the prospective KCSG-LU15-09 trial for this group^33^, a time substantially shorter than the 18.9 months observed for patients with classical EGFR mutations^12^.

To date, whether the efficacy of osimertinib and afatinib differs for atypical EGFR group 1 mutations remains unclear, and further investigations (e.g., a head-to-head comparison) are urgently required. Overall, most clinical data exist for afatinib from clinical trials, compassionate use and early-access programs, as well as non-interventional trials and case reports^32^, but their utility in clinical decision-making is questionable because of the one-sided focus on afatinib. Whereas afatinib has received an extension of the FDA label for patients with NSCLC bearing L861Q, G719X and S768I mutations, no specific approval for patients with atypical EGFR currently exists in Europe. The EMA label for most EGFR TKIs is based on “tumors with *activating* EGFR mutations”. The assessment and clinical interpretation requires substantial knowledge of the complexity of activating EGFR mutations. Therefore, molecular tumor boards should guide the therapeutic decision-making process. Despite the problem of low case numbers, the disappointing PFS range of 8.2–10.7 months for different TKIs in clinical trials^31,33^ indicates that atypical EGFR mutations have relatively low oncogenic potential, and affected patients might benefit from additional treatments beyond EGFR TKIs.

### EGFR exon 20 insertions

Patients with NSCLC with EGFR exon 20 insertion mutations are a heterogeneous subgroup that is difficult to treat (the frequency of different exon 20 insertions is depicted in **Figure 3**)^34^. These patients do not benefit from standard TKIs, with some exceptions (e.g., exon20 763_764insFQEV is sensitive to erlotinib, and exon 20 773_774insGHPH is sensitive to afatinib)^30,35,36^, although in the case of osimertinib, dose escalation to 160 mg daily might be beneficial^37^. For most patients, however, platinum-based chemo- or chemoimmunotherapy represents the standard of care, despite overall disappointing ORRs of 19%–25% and a median PFS of only 5–7 months^30,38,39^. Most exon 20 insertions comprise 3–12 base pairs and occur in frame, thus causing an inward shift of the phosphate-binding loop (so-called P-loop) into the drug-binding pocket and eliciting steric hindrance against most TKIs^7^. In contrast, insertions into the C-helix (e.g., the 763_764ins FQEV) display characteristics of classical EGFR mutations with sensitivity to erlotinib^40^. Insertions outside the C-helix can be divided into near loop and far loop insertions.

Recently, improved structural understanding has led to the development of a newer generation of EGFR/HER2 inhibitors, such as poziotinib, which circumvents the steric hindrance preventing other EGFR TKIs from binding the receptor. ZENITH20 is a large phase II multicohort trial investigating the safety and efficacy of poziotinib in patients with NSCLC with EGFR or HER2 exon20 insertions. The ORR in treatment-naïve patients with EGFR exon20ins (cohort 3) was 27.8%, and the PFS was 7.2 months^41^. Notably, the trial raised safety concerns, because 66% of patients experienced grade 3 or 4 toxicity, mostly because of wildtype EGFR inhibition. Other shortfalls of poziotinib are the lack of inhibition of EGFR T790M or C797 variants^42^, and differences in the *in vitro* sensitivity of cells with near loop and far loop insertions. These shortfalls may potentially be overcome by exon 20 insertion-specific TKIs, such as mobocertinib (TAK-788), which irreversibly binds EGFR *via* a covalent modification of the Cys797 residue in the active site^43^. A DCR, ORR and PFS of 86%, 46% and 7.3 months (*vs*. 3.5 months in real world data), respectively^44,45^, led to a breakthrough therapy designation by the FDA in April 2020. In the subsequent phase II extension study (EXCLAIM), platinum pretreated patients achieved an ORR of 28% and a median PFS of 7.3 months^46^. Despite its much lower affinity for wildtype EGFR, the adverse effects of mobocertinib resemble those of classical TKIs, with mainly gastrointestinal and cutaneous toxicity^44^. Mobocertinib is now FDA approved for pretreated patients with advanced-stage NSCLC bearing EGFR exon 20 insertions, and trials comparing mobocertinib and platinum-based chemotherapy as first line treatments are currently ongoing (e.g., EXCLAIM-2 NCT04129502).

Another emerging therapeutic concept for tumors with EGFR exon 20 insertions is antibody-mediated receptor degradation. Amivantamab is a fully human bispecific monoclonal antibody directed against EGFR and MET, which exerts cytotoxic effects *via* receptor degradation and antibody-dependent cytotoxicity through the Fc domain^47,48^. In preclinical models of exon20ins mutant NSCLC, amivantamab has shown better antiproliferative activity than gefitinib, osimertinib or poziotinib^47^. In CHRYSALIS, a phase I trial, amivantamab has been investigated in pretreated patients with advanced NSCLC with EGFR exon20ins, both as a single agent and in combination with platinum-based chemotherapy or with EGFR TKI lazertinib. The ORR for amivantamab monotherapy was 40%, and the PFS was 8.3 months. The most frequent adverse events were rash (72%) and infusion-associated reactions (60%), most of which remained grade 1 or 2^49^. Amivantamab was granted accelerated FDA approval in May 2021, thus making it the first approved therapy for patients with NSCLC with EGFR exon20 insertions after failure of platinum-containing chemotherapy. Interestingly, the efficacy of amivantamab appears to be independent of the type of exon20 insertion. **Table 2** summarizes the clinical outcomes of patients with NSCLC bearing EGFR exon 20 insertion mutations.

**Table 2:** Clinical outcomes of poziotinib, amivantamab and mobocertib in patients with NSCLC bearing EGFR exon 20 insertion mutations

Study	Phase	Patients (*n*)	Drug	Cohorts	ORR	mPFS (months)	Ref.
Zenith20	Phase II	79	Poziotinib	Cohort 3 (treatment-naïve)	27.8%	7.2	^80^
Park et al., 2021 (Chrysalis 1)	Phase I	81	Amivantamab	Pretreated (chemotherapy)	40%	8.3	^49^
Zhou et al., 2021 (NCT02716116)	Phase I/II	114	Mobocertinib	Pretreated (chemotherapy)	28%	7.3	^46^

## The HER2 (ERBB2) receptor

HER2, a well-known proto-oncogene located on the long arm of chromosome 17 (17q21), is composed of an extracellular region, a transmembrane domain and a tyrosine kinase effector domain with a regulatory domain at the C-terminus. The kinase domain shares high structural and functional homology with EGFR^50^. Unlike other ERBB family members, no soluble ligand of HER2 is known to date, and the receptor itself and downstream signaling are activated *via* homo- and/or heterodimerization with other ERBB family members^51^. HER2 is overexpressed in different tumor types, including breast or gastric/gastroesophageal cancer, and overexpression is associated with an inferior prognosis^52^.

### HER2 alterations in NSCLC

HER2 alterations, including HER2 overexpression, gene amplification and gene mutations, are well established oncogenic drivers in NSCLC and are found in approximately 3% of all NSCLC cases, mostly adenocarcinomas^53^. These alterations result in kinase activation; are functionally transforming in lung cancer models; and have been associated with non-smoking status, female sex, younger age, elevated risk of brain metastasis and dismal prognosis^54–56^. Therapeutically, these alterations represent distinct molecular targets^57^. In NSCLC, HER2 protein expression is detected and quantified by tissue-based immunohistochemistry, for which a score of 2+ or higher is defined as HER2 positive^54^. The incidence of HER2 overexpression varies between 2.4% and 38% in NSCLC, and is more often seen in adenocarcinomas^58–60^. Whereas the connection between HER2 amplification (detected by fluorescence *in situ* hybridization) and overexpression (observed e.g., in breast cancer) remains less clear in NSCLC^60^, *HER2* amplification is observed *de novo* in 3% of lung adenocarcinomas^57^ or is acquired in 13% of patients after failure of targeted therapy^61^.

**Figure 4:**
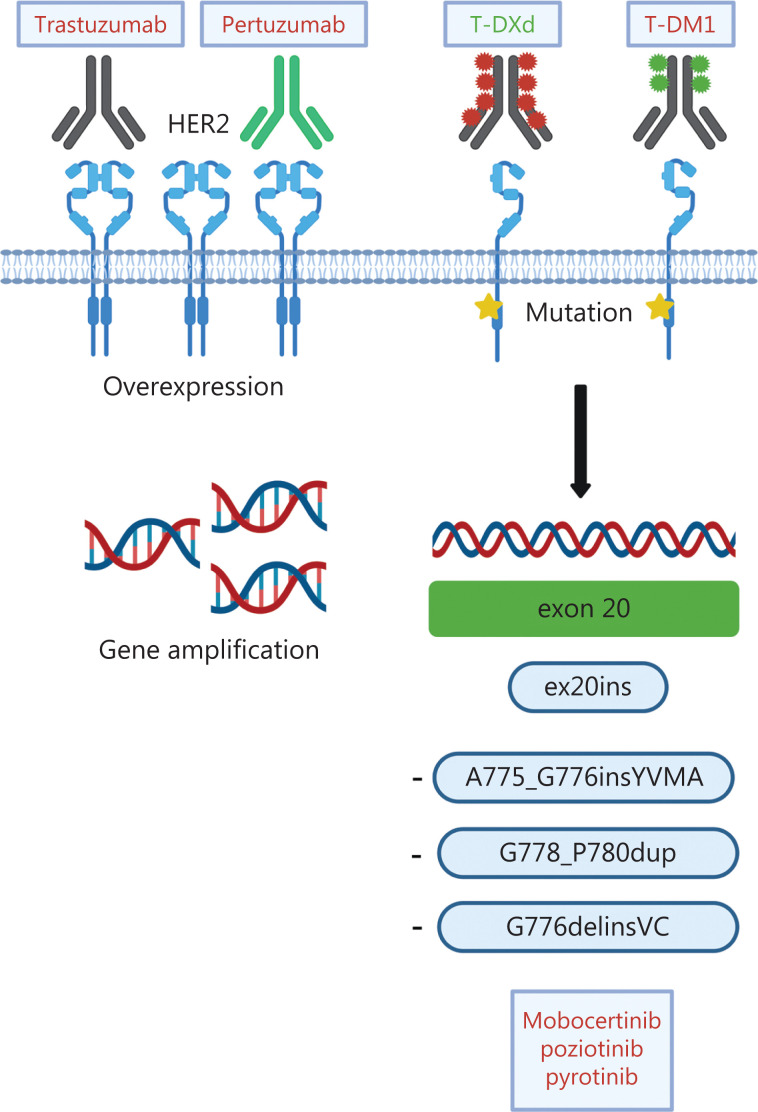
Different modes of HER2 activation and treatment options in NSCLC. In NSCLC, HER2 alterations include HER2 overexpression, HER2 gene amplification and HER2 mutations. Current treatment options consist of the monoclonal antibodies trastuzumab and pertuzumab, the antibody-drug conjugates (ADC) trastuzumab-emtansine (T-DM) and trastuzumab-deruxtecan (TDXd), as well as tyrosine kinase inhibitors targeting HER2 mutations. Approved drugs are depicted in green. Created with BioRender.com.

The discovery that mutations in the kinase domain of HER2 can be detected in ~4% of patients with NSCLC^62^ sparked scientific and clinical interest in targeting HER2 in lung cancer, because HER2 inhibitors (e.g., trastuzumab) had previously been demonstrated to be ineffective in patients with amplification and/or overexpression^63^. Most HER2 mutations (as many as 96%) occur in-frame within exon 20 and resemble EGFR exon 20 mutations^53^. In contrast to the inherently more heterogeneous EGFR exon 20 mutations, 80% of HER2 exon 20 mutations are insertions/duplications of the 4 amino acids YVMA at codon 775 (A775_G776insYVMA)^64,65^. Beyond these, several point mutations, such as p.L755S and p.G776C^53^, as well as rare mutations affecting the transmembrane and juxtamembrane domain, have been described^66^. Numerous strategies to target HER2 molecular alterations in NSCLC have been tested, including conventional chemotherapy, anti-HER2 antibodies, TKIs and, more recently, antibody-drug conjugates (ADC) (**Table 3** and **Figure 4**).

**Table 3:** Clinical outcomes of patients with NSCLC bearing HER2 mutations

Study	Phase	Patients (*n*)	Drugs	ORR	mPFS (months)	Ref.
Kris et al. 2015	Phase II	26	Dacomitinib	12%	3	^67^
Peters et al. 2018	Retrospective	28	Afatinib	19%	2.9 (TTF)	^71^
Lai et al. 2019	Retrospective	23	Afatinib	13%	6 (DoR)	^72^
Hyman et al. 2018	Phase II	26	Neratinib	3.8%	5.5	^3^
Mazieres et al. 2016	Retrospective	29	Afatinib, neratinib or lapatinib	7.4%	3.4	^70^
Zhou et al. 2020	Phase II	60	Pyrotinib	30%	6.9	^83^
Socinski et al. 2020	Phase II	90	Poziotinib	35.1%	5.5	^79^
Mazieres et al. 2016	Retrospective	58	Trastuzumab + chemotherapy	50%	4.8	^70^
Mazieres et al. 2021	Phase II	45	Trastuzumab, pertuzumab, docetaxel	29%	6.8	^92^
Li et al. 2018	Phase II	18	Trastuzumab-emtansine	44%	5	^94^
Iwama et al. 2022	Phase II	22	Trastuzumab-emtansine	38.1%	2.8	^96^
Li et al. 2022	Phase II	91	Trastuzumab-deruxtecan	55%	9.3	^106^

### Tyrosine kinase inhibitors for HER2-altered NSCLC

In contrast to the success of EGFR TKIs for EGFR mutant NSCLC, dual EGFR/HER2 (lapatinib) or irreversible pan-HER TKIs (afatinib, dacomitinib and neratinib) have only limited activity against HER2-mutant refractory NSCLC, with response rates ranging between 0% and 19%^67–69^. In the retrospective EUHER2 study, 11 patients with HER2 exon20ins NSCLC received afatinib, which achieved only modest activity (ORR 18.2% and median PFS 3.9 months)^70^. Similar results have been reported by Peters et al.^71^ from the compassionate use program of afatinib: a median TTF of only 2.9 months and an ORR of 19% were observed, but subgroup analyses revealed that the HER2 A775_G776insYVMA mutation may be a predictor of a longer treatment response, as supported by retrospective analyses^72^. Of note, another observational study has reached an opposite conclusion: patients with the A775_G776insYVMA insertion did not respond to afatinib, whereas patients with G778_P780dup and G776delinsVC mutations derived the greatest benefit from afatinib (ORR 40% and PFS 7.6 months)^73^. These contradictory findings may be due to differences in the co-mutational patterns.

Patients with NSCLC with HER2 exon20ins have not been found to derive a meaningful benefit from lapatinib, a dual EGFR/HER2 inhibitor, as a single agent or in combination with pemetrexed^74,75^. However, in these studies, patients were not stratified by HER2 status. Additionally, neratinib, an irreversible inhibitor of EGFR, HER2 and HER4, has demonstrated a disappointing ORR of only 3.6% as a single agent in the SUMMIT phase II basket trial^3^. Whereas a combination of neratinib with the mTOR inhibitor temsirolimus increased the ORR to 33% in a phase I trial, the subsequent phase II trial exhibited an inferior ORR of 19%^76,77^. Ultimately, the irreversible pan-ERBB inhibitor dacomitinib had limited efficacy^67^, with an ORR of 12% in a phase II study in which none of the 4 patients with HER2 amplification responded.

In light of these disappointing results, better understanding of the structural changes induced by exon20 insertions in HER2 led to the development of new TKIs, such as mobocertinib, poziotinib and pyrotinib. Mobocertinib suppresses tumor growth in patient-derived xenograft models of NSCLC with HER2 exon 20 A775_G776insYVMA^78^, whereas in genetically engineered mouse models with the same mutation, responses are only partial and transient. Sustained complete remission has been achieved with mobocertinib in genetically engineered mouse models with HER2 p.G776del insVC^78^. A clinical trial of mobocertinib in patients with NSCLC bearing exon20 mutations in EGFR or HER2 is currently underway but has to date only reported results for the EGFR cohort^46^.

Poziotinib is a covalent pan-HER inhibitor, and its smaller molecule size than that of afatinib results in better binding to the sterically confined binding pocket. Poziotinib has achieved a PFS of 5.5 months in cohort 2 of the ZENITH20 trial, but high rates (up to 90%) of dose interruptions might limit its clinical applicability^79^. Switching from a once-daily to a twice-daily dosing regimen significantly improves the toxicity profile of poziotinib^80^. In early 2022, the FDA accepted a new drug approval application for poziotinib for patients with NSCLC with HER2 exon20ins.

Pyrotinib is a novel irreversible TKI of HER1, 2 and 4, which has shown activity in pretreated HER2 positive breast cancer^81^. Clinical data for NSCLC remain scarce, but pyrotinib has higher antitumor activity than afatinib in HER2-mutant organoid systems^82^, and a phase II trial enrolling chemotherapy-pretreated patients with HER2-mutant advanced lung adenocarcinoma has indicated promising activity, with a PFS of 6.9 months^83^. Currently, the ongoing phase III PYRAMID-1 trial (NCT04447118) is comparing pyrotinib with docetaxel in patients with NSCLC with HER2 exon 20 mutations as a second line treatment.

Another interesting agent is tarloxitinib, the prodrug of the pan-HER-inhibitor tarloxitinib-e, which is released under hypoxia, thus increasing drug concentrations in the hypoxic tumor microenvironment^84^. *In vivo* studies have suggested antitumor activity for this drug against EGFR and HER2 mutations. Moreover, a phase II trial, which is currently recruiting patients with NSCLC bearing EGFR/HER2 mutations, has reported some early signs of clinical activity for the HER2 exon 20 p.A775_G776insYVMA mutation^84^.

### Monoclonal antibodies for HER2-altered NSCLC

Trastuzumab, a monoclonal humanized IgG1 antibody, binds the extracellular domain of HER2 and blocks its dimerization with other ERBB family members (**Figure 4**). Trastuzumab is approved for breast and gastroesophageal cancer with HER2 overexpression and/or amplification^85^. Cappuzzo and colleagues published the first successful report of treatment of a patient with a HER2 mutant NSCLC with trastuzumab in 2006^86^, and retrospective data from the European EUHER2 trial cohort of relapsed NSCLC with HER2 mutations support this approach, with an observed ORR of 50% and a PFS of 4.8 months in the trastuzumab plus chemotherapy group^70^. However, subsequent studies investigating the combination of trastuzumab plus chemotherapy have found disappointing results in patients with NSCLC, regardless of HER2 amplification or overexpression^87–89^. Whereas pertuzumab, a dimerization inhibitor, yielded disappointing findings with no treatment responses observed^90^, dual HER2 blockade with pertuzumab and trastuzumab in the phase II MyPathway basket trial achieved ORRs of 21% and 13% for patients with HER2 mutations and amplification/overexpression, respectively^91^. The IFCT-1703 R2D2 trial investigating the combination of trastuzumab, pertuzumab and docetaxel has achieved an ORR of 29% and a PFS of 6.8 months^92^.

### Antibody-drug conjugates for HER2-altered NSCLC

The landscape of HER2 directed therapies dramatically changed with the introduction of ADCs, such as trastuzumab-emtansine (T-DM1) and trastuzumab-deruxtecan (T-DXd). ADCs combine the specificity of a monoclonal antibody with the cytotoxicity of chemotherapy (**Figure 4**). In T-DM1, trastuzumab is stably linked to DM1, a derivative of the microtubule inhibitor *maytansine*^93^. In a phase II basket trial, patients with HER2-mutant NSCLC treated with T-DM1 had a median PFS of 5 months^94^, and a clinical benefit (ORR 44%) was subsequently confirmed in an exploratory phase II trial^95^. Additionally, a Japanese study has reported an ORR of 38.1% and a PFS of 3.5 months^96^. Treatment predictors, however, remain undefined, because HER2 overexpression determined by immunohistochemistry was insufficient to predict response to T-DM1^97^.

The second ADC, trastuzumab-deruxtecan (DS-8201a), consists of trastuzumab conjugated with deruxtecan, a novel topoisomerase I inhibitor (**Figure 4**)^98^. A sophisticated linker-payload system enables conjugation of 8 molecules of deruxtecan per antibody (drug-to-antibody ratio 8:1), and the additional high membrane permeability enables targeting of cells with low HER2 expression^99^. In heavily pretreated patients with breast cancer, T-DXd has achieved convincing responses, with a median duration of responses ranging from 10.4 to 20.8 months, depending on HER2 expression levels^100,101^. The DESTINY-Breast04 study has recently established T-DXd as the standard of care for patients with pretreated HER2-low breast cancer, in whom the ADC, compared with standard chemotherapy, improved the PFS by 4.8 months^102^. T-DXd has also been found to significantly improve treatment outcomes for pretreated HER2 positive gastro-esophageal carcinomas^103^. A major advantage of T-DXd is that it is internalized by HER2-mutant NSCLC cells independently of HER2 expression levels. Therefore, it can overcome resistance to other anti HER2 agents^104^.

In the first-in-human clinical trial, T-DXd induced tumor regressions in 11/18 patients with NSCLC; notably, all 8 patients with HER2 exon 20ins responded^105^. The subsequent DESTINY-Lung01 trial confirmed that T-DXd induces durable responses in previously treated patients with HER2-mutant NSCLC, and 55% of patients refractory to prior standard treatment had an objective response lasting for a median of 9.3 months. The median PFS and OS were 8.2 and 17.8 months, respectively^106^. Importantly, responses were observed across different HER2 mutational subtypes, independently of HER2 amplification or overexpression. A major adverse effect of T-DXd, however, was the increased risk of interstitial lung disease, which affects approximately one-quarter of patients and is more frequent at higher T-DXd doses. In August 2022, T-DXd was granted accelerated approval by the FDA for treatment of patients with advanced HER2-mutant NSCLC who have received prior systemic therapy. T-DXd thus became the first drug approved specifically for HER2-mutant NSCLC.

### Chemo- and immunotherapy for HER2-altered NSCLC

Chemo- or chemo-immunotherapy remains the first line treatment of choice for patients with NSCLC with HER2 alterations, because these treatment modalities have limited activity in advanced treatment lines^70^. Nevertheless, the specific importance of HER2 alterations in the efficacy of conventional chemotherapy remains relatively unclear. Although HER2 overexpressing NSCLC cell lines exhibit intrinsic resistance to chemotherapy, particularly to platinum containing regimens^107^, in many prospective clinical trials including patients with NSCLC, HER2 amplification or overexpression has not correlated with response to conventional chemotherapy^108^. Wang et al.^109^ have reported inferior outcomes in patients with HER2 mutations than those with ALK/ROS1 rearrangements, after treatment with pemetrexed. Li et al.^110^ have reported that patients with HER2 mutations respond better to pemetrexed-based chemotherapy than patients with HER2 wildtype. In the EUHER2 cohort, chemotherapy achieved an ORR of 43.5% and a PFS of 6 months in the first line and 10% and 4.3 months, respectively, in the second line^70^. Additionally, in the retrospective IMMUNOTARGET registry trial, patients with NSCLC with HER2 exon 20 mutations treated with immune checkpoint inhibitor (ICI) monotherapy had an ORR of 7% and a median PFS of 2.5 months^111^. Another retrospective analysis from the German National Network Genomic Medicine (nNGM) has provided efficacy data for ICI/platinum-doublet combinations in patients with HER2mut NSCLC. The ORR was 52%, and the mPFS was 6 months. In the second line, the ORR and PFS with ICI monotherapy were 16% and 4 months, respectively^112^.

## The HER3 (ERBB3) receptor

HER3 is a membrane bound protein encoded by the *ERBB3* gene. Despite not being oncogenic itself, e.g., when overexpressed^113^, HER3 plays an important role during tumorigenesis^114,115^. Among other receptors (e.g., FGFR)^116^, HER3 is an important mediator of signaling rebound kinetics after drug interventions in lung cancer (so-called “kinome reprogramming”)^115,117^. The residual kinase activity of HER3, which has been estimated to be 1000-fold weaker than that of EGFR, prevents HER3 from forming homodimers^114,118^. Thus, HER3 requires hetero-dimerization with other ERBB family members (preferentially with EGFR or the ligand-binding impaired HER2, and with lower affinity for HER4) or non-ERBB family receptors, including the mesenchymal-epithelial transition (MET) factor receptor and the fibroblast growth factor receptor 2 (FGFR2) to induce C-terminal phosphorylation and activation of oncogenic downstream signaling e.g., *via* the PI3K/AKT, MAPK and JAK/STAT pathways^119–123^.

### Treatment options for HER3

Owing to its low kinase activity, therapeutic antibodies rather than small molecule inhibitors are currently considered the optimal therapeutic approach to inhibit HER3. Although anti-HER3 monoclonal antibodies showed therapeutic efficacy in preclinical models almost a decade ago^124–126^, no HER3-targeting monoclonal antibody has been approved to date. A major shortfall is the current lack of predictive biomarkers^126^. The addition of a cytotoxic payload to the anti-HER3 antibody patritumab enhances target cell killing, and recent promising phase I data may lead to the drug’s approval in some patient cohorts. Prospectively, therapeutic targeting of HER3 with monoclonal antibodies could also allow for non-invasive imaging and hence more in-depth characterization of tumor biology^127^.

### Intrinsic and drug induced HER3 expression in NSCLC

Depending on tumor stage, as many as ~80% of NSCLCs have been estimated to express HER3^128–131^. Furthermore, circulating tumor cells in patients with NSCLC, mediators of metastatic spread and predictors of an inferior prognosis express HER3^129,132^. Ultimately, apart from intrinsic HER3 expression, treatment with EGFR or MAPK pathway inhibitors elicits the phenomenon of “dynamic kinome reprogramming,” i.e., the upregulation of receptor tyrosine kinases after targeted inhibition of selected kinases, which is mediated partly *via* HER3^117^.

In the case of EGFR TKIs (e.g., gefitinib, erlotinib, afatinib and osimertinib), which have dramatically changed the treatment landscape for patients with *EGFR*-mutant NSCLC, treatment resistance occurs through on-target and/or bypass resistance mechanisms, the latter of which include HER3-mediated bypass activation in the context of a *MET* amplification^120,125^. However, intrinsic and acquired EGFR TKI resistance are heterogeneous at the intra- and interpatient levels^133^, and, in view of the limited benefit of salvage therapy after progression on EGFR TKIs, innovative treatment concepts, ideally agnostic to the underlying resistance mechanism, are urgently needed. Recently, a phase I dose escalation/expansion study (NCT03260491) has reported a confirmed objective response rate of 39% and a median progression-free survival of 8.2 months for patients with locally advanced or metastatic *EGFR*-mutant NSCLC treated with patritumab deruxtecan (HER3-DXd; U3-1402), an ADC consisting of an IgG1-kappa HER3 monoclonal antibody combined with the topoisomerase I inhibitor deruxtecan, after prior failure of EGFR TKI therapy. Interestingly, responses were agnostic to the EGFR TKI resistance mechanism and were observed across a broad range of HER3 membrane expression levels. The most common grade ≥3 treatment-associated adverse events were hematologic toxicity, with a low discontinuation rate (9%), and treatment-associated interstitial lung disease occurred in 5% of patients^134^. Currently, a phase II study is exploring the safety and efficacy of HER3-DXd as a single agent in *EGFR*-mutant NSCLC after progression on EGFR TKIs and one platinum-based chemotherapy-containing regimen (NCT04619004). In addition, a multi-arm phase I study is investigating whether simultaneous EGFR inhibition with osimertinib translates to enhanced anticancer activity of HER3-Dxd, as has been observed in preclinical models and patients, through increased HER3 target expression and ADC uptake (NCT04676477)^135,136^.

An interesting question in the future will be whether patients with NSCLC might also benefit from HER3-DXd when they are simultaneously treated with immunotherapy or other molecular targeted agents (e.g., direct KRAS G12C, MEK or ALK inhibitors). Preclinical data suggest that increased expression of HER3 and NRG1 contributes to ALK inhibitor resistance^137–139^, thereby supporting a potential sensitizing effect of HER3-DXd to ALK blockade^140^. Furthermore, the continual dependency of *KRAS* mutant NSCLC on upstream receptor tyrosine kinases including HER3, as well as the dynamic kinome reprograming after MAPK pathway inhibition^117^, provides a strong scientific rationale for combining these drug classes with HER3-DXd. This strategy, if successful, may overcome the limitation of high rates of skin and gastrointestinal tract toxicity observed with the combination of dacomitinib, a pan-HER inhibitor, and the MEK1/2 inhibitor PD0325901 in patients with *KRAS* mutant NSCLC^141^. In the ongoing phase I dose expansion study (NCT03260491), the cohort of patients with advanced NSCLC without *EGFR* mutation who received prior platinum-based chemotherapy with or without immune therapy showed promising clinical activity, including patients with other driver genomic alterations, such as *KRAS*, *NRAS*, *ALK* and *ROS*^142^. For all indications, the validation of predictive biomarkers for HER3-DXd will be imperative. In the NCT03260491 trial, a non-significant trend of higher response rates to HER3-DXd in patients with higher baseline HER3 expression has been observed^134^.

### Ligand-mediated HER3 activation through NRG1 gene fusions

HER3-dependent signaling is also activated through auto- and/or paracrine secretion of Neuregulin-1 (NRG1 or heregulin), the main HER3 ligand. In 2014, *NRG1* fusions arising from chromosomal rearrangements were discovered as a potentially actionable oncogenic driver in lung cancer^143^. Since then, multiple fusion partners (e.g., *CD74-NRG1*, *SLC3A2-NRG1* and *VAMP2-NRG1)* have been identified^144^. Although *NRG1* fusions occur at low frequency (<1%) across carcinomas^145^ and in mesenchymal tumors^146^, they are enriched in several cancer types, including oncogenic driver-negative NSCLC, and in mucinous lung adenocarcinomas^147^. Most of the resulting oncoproteins retain the EGF-like domain of NRG1 and thus have been predicted to be functional in activating the ERBB2-ERBB3 heterodimer and downstream signaling, thus supporting a therapeutic paradigm of ERBB2/ERBB3 inhibition^143,144,148^. In NRG1-rearranged lung tumors, preclinical and clinical responses to the pan-ERBB inhibitor afatinib^149–151^ and to HER3-targeting monoclonal antibodies [e.g., seribantumab/MM-121^152^, GSK2849330 (NCT01966445)^153^] have been reported; however, the real-world eNRGy1 global multicenter registry has reported an ORR of only 25% and a median PFS of 2.8 months for afatinib^154^. Furthermore, responses to immunotherapy, chemo-immunotherapy (ORR 20% and 0%, respectively) and chemotherapy (ORR ~13%) have been disappointing^154^. Zenocutuzumab (MCLA-128), a HER2/HER3 bispecific antibody, is efficacious in preclinical models and in human tumors driven by NRG1 gene rearrangements. *In vitro* data suggest that zenocutuzumab, apart from blocking oncogenic signaling, elicits antibody-dependent cellular cytotoxicity, thereby engaging the patient’s immune system to kill cancer cells^155^. Zenocutuzumab was granted orphan drug designation by the U.S. FDA for the treatment of patients with pancreatic cancer in 2020, and it received a fast track designation for patients with metastatic solid tumors bearing *NRG1* gene fusions after progression on standard therapy in 2021. In the currently ongoing phase II eNRGy trial (NCT02912949), 35% of 46 evaluable patients with NRG1 fusion-positive NSCLC who had progressed after standard therapy had an objective response to zenocutuzumab (MCLA-128; Zeno). The median duration of response across all tumor entities reached 9.1 months after a median of 2 prior therapies, and the tolerability profile was favorable^156^. At the recommended phase II dose of zenocutuzumab, most treatment-associated adverse events were grade 1 or 2 (diarrhea 21%, infusion-associated reactions 15% and fatigue 12%), and only 5% of patients experienced grade 3/4 treatment-associated adverse events. These recent eNRGy data suggest that zenocutuzumab may become the first targeted therapy for tumors with *NRG1* fusions, either as a tumor-agnostic option for molecularly defined subtypes or for certain disease types. Important open questions remain regarding how the tumor type, fusion partner of *NRG1* and length of the fusion product affect the efficacy of zenocutuzumab.

### HER3 gene mutations in NSCLC

Whereas ERBB3 mutations are more frequent in other tumor types (~11% in colon and gastric cancers, and ~6% in bladder and small bowel cancer)^157,158^, they rarely occur in lung cancer (1%–2%), and the prevalence is potentially higher in some NSCLC subsets e.g., squamous cell carcinoma^159,160^. Mutations affect the extracellular or tyrosine kinase domain of HER3; the latter has the highest oncogenic potential^157,158^ and requires kinase active ERBB2 to activate oncogenic signaling^158,161^. Consequently, HER-targeted inhibitors, such as the pan-ERBB inhibitor afatinib or the HER2-inhibitor pertuzumab, potently suppress mutant HER3 activity^161^. Additionally, LUX-Lung 8, which included patients with stage IIIB/IV lung squamous cell carcinoma progressing after platinum-based chemotherapy, has reported a trend toward better PFS [HR = 0.52 (0.16–1.72)] for patients with *ERBB3* mutations treated with afatinib rather than erlotinib; however, the number of patients in this subgroup was small (*n* = 15), and other treatment comparators were lacking^160^. ERBB3 mutations affecting the germline are also involved in the pathogenesis of familial NSCLC (*ERBB3* mutation c.1946 T>G: p.Iso649Arg)^162^ and are considered an important mediator of immune escape in gallbladder cancer^163^. Despite the apparent effects of HER3 mutations on lung cancer biology, no large-scale clinical trials have been conducted to validate a specific treatment strategy for *ERBB3*-mutant lung tumors, in contrast to activating *EGFR* mutations. Therefore, further studies are needed to fully elucidate the biology of ERBB3 mutants, their predictive and prognostic value, and their contributions to acquired resistance against other treatment modalities e.g., EGFR TKIs.

## The HER4 (ERBB4) receptor

The *ERBB4* gene encodes the 180-kDa transmembrane receptor HER4, which is activated through binding of several ligands, including the neuregulins, heparin-binding epidermal growth factor (HB-EGF), betacellulin and epiregulin, and subsequent hetero-dimerization with HER2^164^. Although the effects of HER4 expression remain controversial in human NSCLC, some studies have claimed that HER4 confers metastatic potential, inferior prognosis and resistance to chemotherapy^165–170^. In concordance with these claims, HER3/HER4 inhibition with an NRG1-targeted monoclonal antibody has been found to inhibit primary tumor growth and enhance the magnitude and duration of chemotherapy response in preclinical models, and to prevent disease relapse^171^. In addition to HER4 overexpression, activating *ERBB4* gene mutations affecting the tyrosine kinase or extracellular domain with oncogenic potential have been reported in ~1%–5% of NSCLCs^160,168,172,173^. Interestingly, these mutations occur in all functional subdomains of the HER4 protein, with no hotspot mutations. This finding is in contrast to lung cancer-associated *EGFR* mutations, most of which typically target few sites in the kinase domain (**Figure 2**). Additionally, rare *ERBB4* fusions with different fusion partners have been described in NSCLC^174,175^. All these fusion proteins retain the full ERBB4 kinase domain and lead to dimerization of 2 HER4-containing fusion proteins and subsequent aberrant activation of the HER4 kinase domain^174^. Although systematic investigations are lacking to date, in a patient with a lung squamous cell carcinoma bearing a missense mutation in the ERBB4 gene (p.Arg847His), afatinib showed some efficacy, but the tumor also carried an *EGFR* amplification^176^. Additionally, in the LUX-Lung 8 clinical trial, which included patients with stage IIIB/IV lung squamous cell carcinoma progressing after platinum-based chemotherapy, patients with *ERBB4* mutations showed a trend toward better PFS [HR = 0.21 (0.02–1.94)] and OS [HR = 0.22 (0.05–1.04)] when treated with afatinib than erlotinib; however, the number of patients in this subgroup was small (*n* = 14), and other treatment comparators were lacking^160^. Therefore, studies on the prevalence of HER4 genomic aberrations, their effects on tumor biology and treatment response to targeted agents (e.g., ~21% of TKI-sensitive *ERBB*-mutant cancer cell lines of different tumor entities exhibit ERBB4 deletions^177^) and conventional chemotherapy, as well as on optimal treatment modalities, should be systematically performed in patients with NSCLC in the future, e.g., in the form of a world-wide registry trial similar to the eNRG trials. **Table 4** summarizes clinical outcomes and trials in patients with NSCLC bearing HER3, HER4 and NRG alterations.

**Table 4:** Clinical outcomes and trials of patients with NSCLC bearing HER3, HER4 and NRG alterations

Study	Phase	Patients (*n*)	Drugs	NSCLC patient cohort	ORR	mPFS (months)	Ref.
NCT03260491	Phase I	57	Paritrutumab-deruxtecan	Locally advanced or metastatic EGFR mutant, pretreated (TKI)	39%	8.3	^184^
NCT03260491 expansion cohort	Phase I	47	Paritrutumab-deruxtecan	Locally advanced or metastatic without EGFR mutation, pretreated (platinum-based chemotherapy ± immunotherapy)	28%	5.4	^142^
NCT04676477	Phase I	Enrolling	Paritrutumab-deruxtecan + osimertinib	Locally advanced or metastatic EGFR mutant, pretreated (osimertinib)	n.n.	n.n.	^185^
eNRGy1	Global multicenter registry	20	Afatinib	NSCLC with NRG1 rearrangements	25%	2.8	^154^
		15	Chemotherapy	NSCLC with NRG1 rearrangements	13%–14%	4.0-5.8	
		9	Chemoimmunmotherapy	NSCLC with NRG1 rearrangements	0%	3.3	
		5	Immunotherapy	NSCLC with NRG1 rearrangements	20%	3.6	
eNRGy trial (NCT02912949)	Phase II	46	Zenocutuzumab (MCLA-128)	NRG1 fusion-positive NSCLC (pretreated)	35%	n.n.	^186^

## Conclusions and future perspectives

In recent years, NSCLC has provided a prime example of the success of oncogene-targeted therapies. This development can overcome the limited treatment results of different regimens of conventional chemotherapy in the early 2000s for this disease^178^, and has improved treatment outcomes for many subgroups of patients with NSCLC, particularly those with adenocarcinoma. The ERBB family of receptors (including EGFR, HER2, HER3 and HER4) has been identified as an important target in NSCLC, owing to the frequency of genomic alterations in this receptor family. The successful approval of different generations of TKIs for the treatment of patients with NSCLC with common activating *EGFR* mutations has reinforced biochemical, preclinical and clinical efforts to target other driver mutations—a strategy that may be impeded for some alterations, particularly if the target protein is expressed at low levels, or the oncogenic potential of the alteration is low or unknown (e.g., HER4 mutations).

New TKI generations with improved safety profiles and efficacy (e.g., osimertinib for EGFR mutant NSCLC with diminished EGFR wildtype inhibition) will be continually developed by improving the structural understanding of the effects of gene mutations on the protein and increasing the ratio of mutant to wildtype receptor inhibition. To develop new drug classes or rational drug combinations, in-depth knowledge of intrinsic and acquired on- and off-target resistance mechanisms is required, thus indicating the need for broad genomic testing of tumor tissue. More frequent re-biopsies at the time of progression enable the detection of secondary alterations (e.g., RET fusions in patients receiving osimertinib), many of which are currently targetable by a second inhibitor (e.g., selpercatinib). Here, the preferential order would be to perform a liquid biopsy first followed by a tissue biopsy in case of inconclusive results. For EGFR TKIs, an additional tissue biopsy is highly important, because trans-differentiation into small-cell lung cancer histology is currently not reliably detected by liquid biopsy. A high medical need for additional treatment strategies persists for patients with EGFR TKI failure whose tumors do not exhibit other druggable co-targets. One approved option for this situation is a combination of chemotherapy, bevacizumab and atezolizumab, according to the IMPower150 trial^29^. After failure of this regimen, patient outcomes remain dismal.

Overall, the role of immunotherapy for EGFR mutant and other ERBB family altered NSCLC will require more in-depth investigations. An interesting though understudied hypothesis is that EGFR TKIs, beyond inhibiting cancer cell growth and survival, may exert pro-immunogenic/pro-inflammatory effects. This hypothesis is based on 2 observations: (1) the high TKI response rates (ORR = 34%) of tumors with low fractions of EGFR mutant cancer cells (5%)^179^ and (2) the increased intratumoral T cell infiltration detected during TKI treatment^180^. Such observations have prompted clinical trials combining TKIs with IO agents (e.g., the phase Ib TATTON trial), but at the cost of an increased risk of severe immune-associated adverse effects (e.g., interstitial lung disease)^181,182^. These findings of (super)additive toxicity together support an immune-associated effect of EGFR TKIs as part of the mode of action, which might be harnessed for therapeutic interventions.

Beyond the undisputed success of targeted therapies for common EGFR mutations, atypical *EGFR* mutations have remained a therapeutic challenge, and very little is known regarding potential resistance mechanisms and subsequent treatment lines in these patients. Therefore, further research on predictive *in silico* and wet laboratory algorithms will be crucial to determine the best choice of current or future EGFR TKIs.

With the approval of mobocertinib and amivantamab for NSCLC with EGFR exon20ins as well as trastuzumab-deruxtecan (T-DXd) as the first drug specifically approved for HER2-mutant NSCLC, other milestones have recently been achieved. However, understanding of treatment predictors, resistance mechanisms and strategies to overcome resistance to these new drug classes will pose a major challenge. Finally, determining how HER3 (e.g., for example, HER3 targeting antibodies zenocutuzumab and patritumab-deruxtecan) and the historically less characterized receptor HER4 will develop into therapeutic targets should prove interesting.

To select the medication of choice from a plethora of targeted drugs, evidence is increasingly indicating that current and future oncologists must learn how to interpret the genetic characteristics of their patients’ lung cancer with a special focus on the ERBB family of receptors (as well as on other oncogenic drivers, such as ALK, ROS1 or KRAS). Molecular tumor boards are the guiding bodies of experts supporting this decision-making process. In the academic setting, translational units consisting of clinical and pre-clinical units will enable increasing characterization of rare genetic events at an accelerated pace to translate and reverse-translate drug sensitivities and resistance mechanisms, e.g., with the help of 3D microfluidic *ex vivo* cultures or tumor spheroids.
